# Genetic and environmental effects on the morphological asymmetry in the scale‐eating cichlid fish, *Perissodus microlepis*

**DOI:** 10.1002/ece3.1691

**Published:** 2015-09-09

**Authors:** Hyuk Je Lee, Valentin Heim, Axel Meyer

**Affiliations:** ^1^ Lehrstuhl für Zoologie und EvolutionsbiologieDepartment of BiologyUniversity of Konstanz78457KonstanzGermany; ^2^ Department of Biological ScienceCollege of Science and EngineeringSangji UniversityWonju220‐702Korea

**Keywords:** Adaptation, behavioral laterality, mouth asymmetry, narrow‐sense heritability, negative frequency‐dependent selection, phenotypic plasticity

## Abstract

The scale‐eating cichlid fish, *Perissodus microlepis*, from Lake Tanganyika are a well‐known example of an asymmetry dimorphism because the mouth/head is either left‐bending or right‐bending. However, how strongly its pronounced morphological laterality is affected by genetic and environmental factors remains unclear. Using quantitative assessments of mouth asymmetry, we investigated its origin by estimating narrow‐sense heritability (*h* ^*2*^) using midparent–offspring regression. The heritability estimates [field estimate: *h* ^*2*^ = 0.22 ± 0.06, *P *=* *0.013; laboratory estimate: *h* ^*2*^ = 0.18 ± 0.05, *P *=* *0.004] suggest that although variation in laterality has some additive genetic component, it is strongly environmentally influenced. Family‐level association analyses of a putative microsatellite marker that was claimed to be linked to gene(s) for laterality revealed no association of this locus with laterality. Moreover, the observed phenotype frequencies in offspring from parents of different phenotype combinations were not consistent with a previously suggested single‐locus two‐allele model, but they neither were able to reject with confidence a random asymmetry model. These results reconcile the disputed mechanisms for this textbook case of mouth asymmetry where both genetic and environmental factors contribute to this remarkable case of morphological asymmetry.

## Introduction

**Figure 1:**
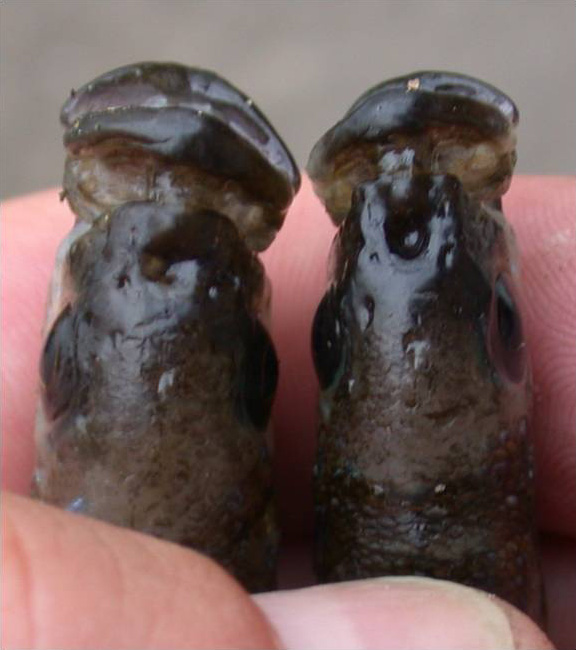
Dorsal view of right‐bending (left) and left‐bending (right) mouth morphs of the scale‐eating cichlid fish, *Perissodus microlepis*, from Lake Tanganyika.

The scale‐eating cichlid fish, *Perissodus microlepis*, from Lake Tanganyika is considered to be a textbook example (Futuyma 2009) for negative frequency‐dependent selection acting on an antisymmetric (dimorphic) trait that causes the two asymmetric forms to be equally frequent (Hori 1993). These scale‐eating cichlids have mouths that tend to be left‐bending (L morph) or right‐bending (R morph) (Fig. 1), although nearly symmetrical mouths occur as well (Kusche et al. 2012). The asymmetric morphs of the scale‐eaters have been hypothesized to be the outcome of an astonishing ecological (trophic) adaptation: Each morph attacks its prey fish primarily from only one side (Hori 1993; Lee et al. 2012). L morphs have a much higher predation success when they attack the right side of their victims, whereas the R morphs attack preferentially and more successfully the left side of their victims (Hori 1993; Takeuchi et al. 2012).

So far, the role of genetic and/or environmental factors contributing to this remarkable morphological laterality remains uncertain (Palmer 2010). Several recent, but contradictory studies on the basis of laterality in *P. microlepis* have been published (Lee et al. 2010, 2012; Stewart and Albertson 2010; Van Dooren et al. 2010; Kusche et al. 2012; Hata et al. 2013). Laterality in this fish was initially suggested to follow a simple Mendelian one genetic locus with two [L and R] alleles model where the R allele is dominant over the L allele (Hori 1993). However, this model was later adjusted: The R allele is both dominant and homozygous lethal (Hori et al. 2007). The latter model has been proposed based on field observations of phenotype (i.e. mouth‐bending morph) frequencies in offspring (*F1*) of broods from breeding pairs of different phenotype combinations. R‐R pairs were observed to raise young in a 1:2 ratio of L:R offspring (which is different from L:R = 1:3 expected under Mendelian inheritance assuming R individuals are heterozygote), L‐R pairs a 1:1 offspring, and L‐L pairs a 1:0 offspring (Hori et al. 2007).

There is some evidence in favor of the genetic model with R dominant and RR lethal (Hori et al. 2007). A single brood from a wild‐caught mouth‐brooding female showed data supporting Hori's model (Hori et al. 2007) and further suggested a putative link between a microsatellite marker (UNH2101) and gene(s) for mouth laterality (Stewart and Albertson 2010). However, an association of a microsatellite locus with mouth laterality was not detected in our previous population genetics study (Lee et al. 2010). Nevertheless, the observed lack of association could still be due to linkage disequilibrium being much narrower in a population sample relative to a pedigree (family) sample because of more accumulated effects of recombination, past admixture, and genetic drift for the former (Lynch and Walsh 1998). Therefore, further studies using multiple families are needed to test the linkage of UNH2101 to laterality gene(s) in *P. microlepis*.

The interpretation of genetic data based on the laterality of the brood compared to their brood‐caring parents from wild is complicated due to apparently prevalent intra‐ and even interspecific brood mixing in this fish (Yanagisawa 1985; Ochi and Yanagisawa 1996). In the previous field studies (Hori 1993; Hori et al. 2007), brood mixing was not taken into account and parentage analyses were not conducted. This might have led to incorrect genetic interpretations as parentage of the young was not tested against their (potential) foster parents.

A recent review (Palmer 2010) pointed out that none of the estimates reported so far fits a single‐locus Mendelian model or random antisymmetry model where mouth laterality of *P. microlepis* is solely environmentally determined, that is, L:R = 1:1 (Palmer 2004). This implies either that the Mendelian models (Hori 1993; Hori et al. 2007) that have been proposed so far are incorrect, or that broods examined are heterogeneous due to brood mixing, or both. Moreover, previous studies [except (Stewart and Albertson 2010)] determined mouth morph of both parents and offspring by visual inspection alone without quantification, although mouth laterality is sometimes quite difficult to judge in adults (Van Dooren et al. 2010; Kusche et al. 2012) and even more difficult to define in juveniles (Stewart and Albertson 2010; Kusche et al. 2012; Lee et al. 2012). Our recent findings of common adult and juvenile individuals with apparently symmetrical mouths and the resulting unimodal (not bimodal) trait distribution of mouth asymmetry (Kusche et al. 2012) further call into question the notion that laterality in *P. microlepis* is determined exclusively by a single genetic locus with two alleles.

Phenotypic plasticity was suggested to play a role in the determination of mouth asymmetry in *P. microlepis* (Van Dooren et al. 2010; Palmer 2011; Lee et al. 2012). In laboratory experiments, mouth asymmetry of adult scale‐eaters allowed to feed on scales of prey fish in their preferred direction significantly increased further toward their initial asymmetry, whereas that of the fish being forced to forage on the nonpreferred side did not (Van Dooren et al. 2010). These findings suggest that handed behavioral foraging preference might induce and contribute to mouth asymmetry of *P. microlepis* through phenotypic plasticity (Palmer 2011; Lee et al. 2012). At this stage, however, a more complete understanding of the genetic and/or environmental bases for mouth laterality in *P. microlepis* would require direct estimation of the laterality of broods from different crosses between/within L and R morphs under standardized laboratory conditions. Furthermore, in the case of field samples of broods, parentage analysis on the offspring against their foster parents is essential to avoid potential bias resulting from the effects of brood mixing.

In this study, we used quantitative measurements of mouth asymmetry to analyze both genetic and environmental influences on morphological laterality in *P. microlepis*. For this, heritability (*h* ^*2*^) of laterality was estimated separately for field (wild‐caught) and laboratory (laboratory‐bred) families. To test the hypothesis that mouth laterality of this species is directed by two alleles at a single Mendelian locus, we also investigated trait distributions and inheritance patterns of laterality and conducted association analyses of the suggested putative marker.

## Materials and Methods

### Samples

Juvenile *Perissodus microlepis* (total length: 7–15 mm) were caught from seven broods with their guarding breeding pairs (i.e., foster parents; 1 L‐L, 5 L‐R, and 1 R‐R pairs whose laterality was determined based on the quantification of their mouth asymmetry; see below) at Toby Veall's lodge (8°37.4′S, 31°12′E) in southern Lake Tanganyika, Zambia, in April 2010. Sampling was carried out by diving with hand nets. The samples were stored in 97% ethanol and transferred to the University of Konstanz. In addition, 65 live young from five broods (3 L‐R and 2 R‐R pairs) were caught and transported to the animal care facility at the University of Konstanz and raised in separate 40‐L and later 200‐L aquaria with *Artemia* nauplii and flake food as diet (Kusche et al. 2012; Lee et al. 2012). As unexpectedly high proportions of extrapair juveniles of other parents were detected within these broods using microsatellite markers (H. J. Lee, V. Heim & A. Meyer, unpubl. ms.), we only used these fish as stock for our breeding experiments.

A total of 229 juvenile fish resulted from 10 broods by breeding these wild‐caught fish in the laboratory. Several fish (e.g., *n *=* *5–8) were initially kept in 200‐L aquaria with approximately even numbers of males and females whose sexes were judged based on their body size (male individuals were typically larger than females at the same ontogenetic stages; H. J. Lee, pers. obs.). *Perissodus microlepis* is socially monogamous and its mating begins with “pair formation.” Thus, aquaria were checked for pair formation almost every day. When pair formation behavior was observed, the pair was immediately transferred to a smaller 120‐L aquarium. Roughly, the first mating from the pair tanks (i.e., the first observation of mouth‐brooding) took more than a week, which ensured there had been no mating before pair isolation. As soon as a female was mouth‐brooding, eggs or hatched larvae were taken from the mouth and incubated in a plastic container (18 × 12 × 12 cm) with aeration. Once the juvenile fish started to swim actively, they were moved to a bigger 40‐L aquarium. Laboratory‐reared *P. microlepis* reached sexual maturity at about 6–9 months of age. There was variation in the number of juveniles per brood used for quantification of mouth asymmetry (see below), ranging from three to 52 (mean number = 23; Table 1), because numbers of initial brood size as well as early mortality rate varied among the broods.

**Table 1:** Phenotype frequencies in *Perissodus microlepis* broods

(A) Wild‐caught 7 families						
Parent phenotypes	L × L (1)		L × R (5)		R × R (1)	
	L:R	L:S:R	L:R	L:S:R	L:R	L:S:R
*F1*	4:0	2:2:0	5:6	5:1:5	7:20	7:2:18
			17:20	13:9:17		
			39:31	29:19:22		
			26:19	19:9:17		
			16:19	11:8:16		
Pooled	**4:0**	**2:2:0**	**103:95**	**77:46:77**	**7:20**	**7:2:18**
Observed ratio	1:0	1:1:0	1.1:1	1.7:1:1.7	1:2.9	3.5:1:9
Expected ratio (Hori et al. 2007)	1:0	–	1:1	–	1:2	–
*P*‐value from a *χ* ^*2*^ *‐*test	–	–	0.32	–	0.41	0.57
Expected ratio (Palmer 2010)	1:1	–	1:1	–	1:1	–
*P*‐value from a *χ* ^*2*^ *‐*test	–	–	0.57	–	0.01	0.03

(B) Laboratory‐bred 10 families			
Parent phenotypes	L × L (2)	L × R (5)	R × R (3)
	L:R	L:R	L:R
*F1*	10:4	4:2	13:15
	8:6	22:29	9:20
		16:20	10:8
		11:13	
		1:2	
Pooled	**18:10**	**54:66**	**32:43**
Observed ratio	1.8:1	1:1.2	1:1.3
Expected ratio (Hori et al. 2007)	1:0	1:1	1:2
*P*‐value from a *χ* ^*2*^ *‐*test	–	0.27	0.09
Expected ratio (Palmer 2010)	1:1	1:1	1:1
*P*‐value from a *χ* ^*2*^ *‐*test	0.13	0.27	0.20

For wild‐caught broods, two independent sets of *χ* ^*2*^ analyses were conducted by (1) considering individuals with negative or positive values of jaw‐bending angle as L or R morphs, respectively, and (2) excluding “symmetrical (S)” morphs that were defined based on the average level of mouth asymmetry observed in another cichlid, *Astatotilapia burtoni* (−1.17° to +1.17°), and then considering the remaining individuals as L or R morphs. For laboratory‐bred broods, only a single set of *χ* ^*2*^ analyses was performed as carried out for (1) described above. The observed phenotype frequencies were tested against expected frequencies under Hori's genetic model (Hori et al. 2007) or antisymmetry model (Palmer 2010). Pooled phenotype frequencies of *F1* according to the parent phenotype combinations are shown in bold. Note that none of *χ* ^*2*^ tests were statistically significant after the sequential Bonferroni correction applied for multiple testing.

### Quantification of mouth asymmetry

**Figure 2:**
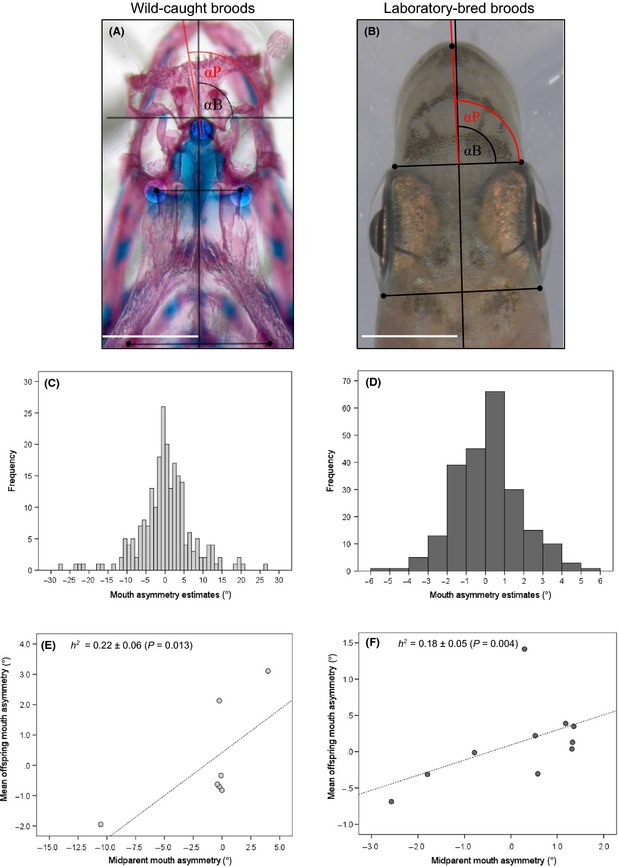
Methods for the quantification (A, B), frequency distributions (C, D), and heritability (*h* ^*2*^) estimates (E, F) of mouth asymmetry in wild‐caught and laboratory‐bred *Perissodus microlepis*. Left panel: wild‐caught broods; right panel: laboratory‐bred broods. (A, B) Vertical black lines designate the “anteroposterior” axis of the skull or head, and red lines denote “jaw‐bending” (A) or “mouth‐bending” (B) axes determined by the prolongation of the premaxillary symphysis (A) or the tip of snout (B) (see detailed descriptions in Materials and Methods). The difference in angles (°) [*α*B − *α*P] was calculated between the two axes and used for jaw/mouth asymmetry estimates. White bars in (A) and (B) represent a scale of 1 and 3 mm, respectively. A continuous and unimodal trait distribution (C, D) and the field and laboratory *h* ^*2*^ estimates (±SE [standard error]; E, F) suggest the genetic and environmental bases of mouth laterality in this species.

For wild‐caught broods of *P. microlepis*, we performed parentage analyses on each of the seven families using six microsatellite loci, including UNH2101 that was suggested to be a putative marker linked to gene(s) for mouth laterality (Stewart and Albertson 2010), to exclude “extrapair” juveniles of other parents within the broods. The six microsatellite loci genotyped were highly polymorphic (the number of alleles per locus ranging from 8 to 15; mean number = 11, mean expected heterozygosity [*H*_E_] = 0.787, mean observed heterozygosity [*H*_O_] = 0.834), and the exclusion probability of both cases of one parent known and neither parent known was 99.6% and 96.7%, respectively (H. J. Lee, V. Heim & A. Meyer, unpubl. ms.). Parentage analyses were conducted using FAP 3.6 (Taggart 2007) based on the exclusion principle where one allele should at least be shared between a parent and an offspring at a codominant microsatellite locus under Mendelian inheritance. After excluding the extrapair juveniles via paternity analyses with microsatellite markers (average among the 7 broods = 41%; H. J. Lee, V. Heim & A. Meyer, unpubl. ms.), the heads of the remaining, genetically assigned “descendant” juvenile specimens (*n *=* *231), which ranged from four to 70 per brood (mean number = 33; Table 1), were cleared and double‐stained using a standardized method (Walker and Kimmel 2007). These stained samples were photographed individually from a dorsal view using a Zeiss Axiophot 2 digital imaging system mounted to a M2 Bio stereomicroscope (Zeiss, Germany). Mouth asymmetry was then quantified by estimating “jaw‐bending” orientation in angles (°) (Fig. 2A). For this measurement, two axes were defined on the skull in each photograph: (1) “anteroposterior” axis of the skull by drawing a straight line through middle points of the two line segments between two paired landmarks digitized on each of the anterior endpoints and posterolateral points of the neurocranium, and (2) “jaw‐bending” axis by drawing a line between the premaxillary symphysis and the posterior midpoint of the premaxillary bone (Fig. 2A). The difference in angles (°) between the two axes (i.e., *α*B – *α*P in Fig. 2A) was then measured in ImageJ 1.46r (http://imagej.nih.gov/ij). Repeatability (*r*) of this measurement technique was assessed using two independent photographs of the same individuals (*n *=* *20), and it was found to be very high (*r *=* *0.96). These repeated measurements were carried out blind (i.e., with no reference to the original measurements). Using the same method, we further estimated mouth asymmetry in juveniles (*n *=* *21) of another African cichlid fish, *Astatotilapia burtoni*, from a single brood with a similar size as the young *P. microlepis*, which are deemed to have symmetrical mouths, to contrast their level of asymmetry with *P. microlepis*. Mouth asymmetry of 14 parental fish for the seven broods, which were kept in 97% ethanol, was also quantified as carried out for the juveniles (but without clearing and staining their heads). The tip of the snout and the anterior‐most and posterior‐most points of the eye sockets were used as reference points to define a “mouth‐bending” axis and an “anteroposterior” axis, respectively, in order to measure “mouth‐bending” orientation in angles (°) (see Fig. 2B).

When laboratory‐bred juveniles of 10 broods were at 2–6 months of age, each live fish was photographed from a dorsal view in a standardized upright position using a Zeiss Axiophot digital microscope (Zeiss, Germany) (Fig. 2B) (Kusche et al. 2012; Lee et al. 2012). Age (body size) was previously found to have no significant effects on levels of mouth asymmetry (Kusche et al. 2012). The 20 parents of these 10 broods were also photographed from a dorsal view using a digital camera. Mouth asymmetry of the juveniles and parents was then estimated by measuring “mouth‐bending” orientation as shown in Fig. 2B. Repeatability (*r*) of this technique was also calculated (*n *=* *20) using repeated, but blind measurements carried out from two independent photographs and it was observed to be lower than that of jaw‐bending orientation for wild‐caught broods, but still fairly high (*r *=* *0.80).

### Data analysis

Dip statistic (Hartigan and Hartigan 1985) was used to test whether variation in mouth asymmetry of *P. microlepis* follows a bimodal or unimodal trait distribution in wild‐caught as well as laboratory‐bred fish, as performed in previous studies (Van Dooren et al. 2010; Kusche et al. 2012). This statistic is a specific test for a unimodal distribution, which has been used for testing any presence of antisymmetry (Van Dooren et al. 2010; Kusche et al. 2012). Also, the mean of mouth asymmetry was calculated and tested for a departure of the mean from zero using one‐sample *t*‐test. Only offspring individuals (wild‐caught fish: *n *=* *231; laboratory‐bred fish: *n *=* *229) were included in these analyses.

To test whether phenotype (mouth morph) frequencies in offspring of parents of different phenotype combinations are consistent with Hori's genetic model (Hori et al. 2007) or the random antisymmetry model (Palmer 2010), a *χ* ^*2*^ goodness‐of‐fit test was applied after pooling data according to the determined parent phenotypes (e.g., L‐L, L‐R, and R‐R pairs). For wild‐caught broods, two sets of independent *χ* ^*2*^ tests were performed by (1) grouping individuals with negative or positive values of jaw‐bending angle as L or R morphs, respectively, and (2) excluding “symmetrical (S)” morphs that were defined based on the average level of mouth asymmetry observed in another cichlid, *Astatotilapia burtoni* (−1.17° to +1.17°), and then considering the remaining individuals as L or R morphs. However, for laboratory‐bred broods, only a single set of *χ* ^*2*^ tests was conducted as carried out for the first method (1) used for wild‐caught broods (see above). Six of the 229 laboratory‐bred fish showed mouth‐bending angles equal to zero, and those were therefore omitted from these analyses. The observed phenotype frequencies were tested against frequencies expected under either hypothesis of the single‐locus two‐allele model with R dominant and RR lethal (Hori et al. 2007) or the antisymmetry model (Palmer 2010).

Association analysis was carried out at the family level to test the linkage of UNH2101 to gene(s) for mouth laterality. Only five of the seven wild families were investigated due to small sample sizes for the other two families (L‐L pair: *n *=* *4; L‐R pair: *n *=* *11; see Table 2). A *χ* ^*2*^ test was conducted to analyze whether observed genotype frequencies at UNH2101 significantly differ from genotype frequencies expected under Mendelian inheritance for each morph. *P*‐values were calculated according to Fisher's exact tests. Two separate analyses (e.g., with/without considering the scored S morphs) were conducted as carried out for the investigations on inheritance patterns of mouth laterality in wild‐caught broods (see above). The significance levels of every *χ* ^*2*^ tests were adjusted for multiple testing using a sequential Bonferroni correction (García 2004).

**Table 2:** Association analyses in five wild‐caught *Perissodus microlepis* families of alleles at a microsatellite locus UNH2101 that was suggested to be linked to gene(s) for mouth laterality (Stewart and Albertson 2010)

Parental phenotypes; genotypes	Morphs	Offspring frequencies of different microsatellite genotypes				*n*	*χ* ^*2*^	df	*P*
		161/161	161/179				2.01 (2.35)	1	0.33 (0.18)
♂: R; 161/161	L	5 (5)	1 (1)			6 (6)			
♀: R; 161/179	R	8 (7)	8 (8)			16 (15)			
	*n*	13 (12)	9 (9)			22 (21)			
		153/153	153/175	175/175			1.14 (0.88)	2	0.57 (0.73)
♂: L; 153/175	L	5 (4)	8 (5)	4 (4)		17 (13)			
♀: R; 153/175	R	3 (3)	11 (9)	6 (5)		20 (17)			
	*n*	8 (7)	19 (14)	10 (9)		37 (30)			
		161/175	169/175				0.05 (0)	1	1.00 (1.00)
♂: R; 175/175	L	17 (11)	12 (10)			29 (21)			
♀: L; 161/169	R	15 (10)	12 (9)			27 (19)			
	*n*	32 (21)	24 (19)			56 (40)			
		161/161	161/179	161/175	175/179		1.61 (1.43)	3	0.66 (0.70)
♀: L; 161/175	L	8 (6)	8 (6)	6 (3)	3 (3)	25 (18)			
♂: R; 161/179	R	3 (3)	5 (4)	4 (4)	4 (4)	16 (15)			
	*n*	11 (9)	13 (10)	10 (7)	7 (7)	41 (33)			
		153/161	161/175				3.54 (3.91)	1	0.09 (0.11)
♂: R; 161/161	L	5 (2)	11 (9)			16 (11)			
♀: L; 153/175	R	12 (9)	7 (7)			19 (16)			
	*n*	17 (11)	18 (16)			35 (27)			

Two separate analyses were performed by (1) considering individuals with negative or positive values of jaw‐bending angle as L or R morphs, respectively, and (2) excluding “symmetrical” morphs that were defined based on the average level of mouth asymmetry observed in another cichlid, *Astatotilapia burtoni* (−1.17° to +1.17°), and then considering the remaining individuals as either L or R morphs (results shown in parentheses). None of the five families showed significant association between mouth morphs and alleles/genotypes typed at UNH2101. *P*‐values were calculated according to Fisher's exact tests.

Narrow‐sense heritability (*h* ^2^) of mouth asymmetry, that is, the proportion of phenotypic variance accounted for by additive genetic variance, was estimated separately for wild‐caught and laboratory‐bred families by calculating the slope of midparent–offspring regression (Lynch and Walsh 1998). Because the number of offspring per family (i.e., family size) largely varied among the broods, we used “weighted” least‐square regressions in which families of larger size were given more weight to minimize sampling error of the heritability estimate (Lynch and Walsh 1998). Heritability estimates are based on the quantitative genetic model that traits are normally distributed (Lynch and Walsh 1998). The *h* ^2^ estimates of mouth asymmetry in *P. microlepis* were applied because its trait distributions were observed to be unimodal rather than bimodal (Palmer 1996).

The morphological data (e.g. mouth asymmetry estimates) and the microsatellite data used in this study have been deposited in DRYAD, doi:10.5061/dryad.3fp18.

## Results

### Trait distribution of variation in mouth asymmetry suggests its genetic and environmental origins

Variation in mouth asymmetry of *P. microlepis* exhibited a continuous and unimodal trait distribution in both wild‐caught and laboratory‐bred broods (wild broods: dip statistic = 0.0154, *n *=* *231, *P *>* *0.99; laboratory broods: dip statistic = 0.0148, *n *=* *229, *P *>* *0.99; Fig. 2C and D), which would be predicted if it were to follow quantitative genetic model, where not only genetic but also environmental variation plays a role in the determination of mouth asymmetry (Lynch and Walsh 1998). The mean of mouth asymmetry estimates was not significantly different from zero in either group of fish (wild broods: *t *=* *0.383, *df* = 230, *P *=* *0.70; laboratory broods: *t *=* *0.903, *df* = 228, *P *=* *0.37). Degree of mouth asymmetry (i.e., absolute values of jaw‐/mouth‐bending angles) was, however, shown to be 3.7 times greater in wild broods relative to laboratory broods [wild: mean = 4.95 ± 5.08 SD (°); laboratory: mean = 1.34 ± 1.09], possibly due to the fact that different measurement techniques were applied to these two groups of fish. However, it is unlikely that the degree of mouth opening affected the measured angles of jaw/mouth asymmetries causing the differences in the mouth asymmetry estimates between wild‐caught and laboratory‐bred broods, considering the low levels of jaw asymmetry shown in another cichlid fish, *Astatotilapia burtoni*.

### Mouth laterality is unlikely to be determined by a single genetic locus with two alleles

Investigations of phenotype frequencies in offspring (*F1*) from parents of L and R morph combinations revealed no evidence that mouth laterality in *P. microlepis* is governed exclusively by two alleles (L and R alleles) at a single Mendelian locus (Table 1). Although phenotype frequencies in the seven wild‐caught broods did not significantly deviate from the expected frequencies under Hori's genetic model (Hori et al. 2007) (*P *≥* *0.32 in all cases), many juvenile individuals (50 of 231 = 22%) showed a level of mouth asymmetry equivalent to another cichlid fish, *Astatotilapia burtoni* (−1.17° to 1.17°), that is not considered to be asymmetrical, which we would, therefore, consider to be “symmetric (S)” (Table 1A). The high abundance of symmetric individuals in L‐L, L‐R, or R‐R breeding pairs is certainly inconsistent with the single‐locus two‐allele model with R dominant and homozygous lethal (Hori et al. 2007). The observed phenotype frequencies in wild‐caught families did not fit a 1:1 ratio of L:R offspring predicted under the antisymmetry model, either (e.g., R‐R pairs; see Table 1A). However, if a sequential Bonferroni correction were applied to these results, none of the *P*‐values would significantly depart from either Hori's genetic model (Hori et al. 2007) or antisymmetry model (Palmer 2010).

In laboratory‐bred broods, frequencies of L and R morphs even more clearly disagree with the single genetic locus model given the presence of R morphs in offspring from L‐L pairs, although a ratio of L:R offspring in L‐R or R‐R pairs did not significantly differ from the expected ratio (Table 1B). However, the observed phenotype frequencies in laboratory families were not significantly different from phenotype frequencies expected under the antisymmetry model (Table 1B).

### Mouth laterality does not segregate with a putative marker

Family‐level association analyses of wild‐caught *P. microlepis* revealed that mouth laterality did not segregate with alleles/genotypes at the putative marker, UNH2101, that was previously suggested to be linked to gene(s) for the laterality (Table 2). In none of the five families tested, did the observed genotype frequencies of the determined mouth morphs differ significantly from genotype frequencies expected under Mendelian inheritance (Fisher's exact tests; *P *≥* *0.09 in all cases; Table 2), suggesting a lack of association of that locus with laterality. These results are consistent with our previous, population‐level analysis of this locus (Lee et al. 2010).

### Heritability (*h* ^*2*^) estimates of mouth laterality

The laterality of mouth asymmetry in both wild‐caught and laboratory‐bred families was significantly heritable [wild‐caught family: *h* ^*2*^ = 0.22 ± 0.06 (SE; standard error); *P *=* *0.013, laboratory‐bred family: *h* ^*2*^ = 0.18 ± 0.05, *P *=* *0.004] (Fig. 2E and F).

## Discussion

This study demonstrates that variation in mouth asymmetry of the Lake Tanganyikan scale‐eating cichlid fish, *Perissodus microlepis*, does not follow simple Mendelian inheritance. However, our results do suggest that mouth asymmetry represents complex (polygenic or quantitative) variation with a weak genetic basis and a sizable environmental component. Although the number of families used in this study is small (e.g., seven wild‐caught and 10 laboratory‐bred families), the both field‐ and laboratory‐heritability (*h* ^*2*^) estimates suggest that at least some of the phenotypic variance observed in morphological mouth asymmetry can be explained by additive genetic variance (18–22%; see Fig. 2E and F), despite a large environmental variance (78–82%).

Multiple lines of evidence support the hypothesis that mouth asymmetry of *P. microlepis* is unlikely to be determined exclusively by two alleles at a single genetic locus, as previously thought (Hori 1993; Hori et al. 2007; Stewart and Albertson 2010). The frequent occurrences of “symmetric” morph (50 of 231 individuals = 22%) in wild‐caught juvenile *P. microlepis* and the resulting unimodal (not bimodal) trait distribution of mouth asymmetry call the previous hypothesis of the single‐locus two‐allele model into question. In fact, our results are consistent with a recent study (Kusche et al. 2012) that also found a continuous and unimodal (and nonplatykurtic) distribution of mouth asymmetry in adult as well as juvenile *P. microlepis*, which actually contradicts a more recent study (Hata et al. 2013) that found distinct antisymmetry in adult *P. microlepis* from Kasenga Point (8˚43′S, 31˚08′E) on the southern tip of Lake Tanganyika. Based on the shape of trait distribution of mouth asymmetry, that study (Kusche et al. 2012) argued that both genetic and environmental factors play a role in the determination of mouth laterality.

Moreover, phenotype frequencies in *F1* broods do not support the previous notion that mouth laterality in *P. microlepis* follows Mendelian inheritance (Hori et al. 2007). If that hypothesis were true, offspring of R (or S) morphs would not be expected from L‐L breeding pairs, which was the case for both of the L‐L pairs from laboratory‐bred families and for the L‐L pair from wild‐caught families (see Table 1). However, in both wild and laboratory families, the observed phenotype frequencies of offspring in either L‐R or R‐R pairs were found not to be significantly different from those expected under Hori's genetic model (Hori et al. 2007). Random antisymmetry model where there would be a 1:1 ratio of L:R offspring in any of parent phenotype combinations is not rejected by our data, either (see Table 1B). It is unlikely, but possible that differences in mortality rates among the broods might affect phenotype frequencies in the offspring of different families reared in the laboratory. Finally, our paternity analyses revealed an unexpected high percentage of extrapair juveniles among field‐collected broods (41%; H. J. Lee, V. Heim & A. Meyer, unpubl. ms.), which means that prior estimates of offspring phenotype frequencies based solely on field broods (Hori 1993; Hori et al. 2007) may not be reliable.

The observed lack of association between mouth morph and allele/genotype typed at UNH2101, the putative marker suggested to be linked to the “laterality gene(s),” further questions the genetic model of Hori et al. (2007). Our findings differ from a recent study (Stewart and Albertson 2010) that suggested that this locus segregates with mouth laterality in *P. microlepis*, and are congruent with our previous work (Lee et al. 2010) that revealed no evidence for linkage between UNH2101 and gene(s) for laterality at the population level. The discrepancy between the results in the current study and those of some previous investigations (Stewart and Albertson 2010) may be possibly due to sampling error caused by the use of only a single family in Stewart and Albertson (2010). It might alternatively be due to differences in the association of the putative marker with mouth morph among local populations owing to “population stratification” (Freedman et al. 2004). Differences in allele frequencies among populations might also be due to differences in ancestry, particularly given the reported detectable population structure at a small geographic scale of less than 10 km (Lee et al. 2010). Both studies [(Stewart and Albertson 2010) and the current study] used a similar measurement technique for the determination of mouth laterality – estimating jaw‐bending angle of the “upper” jaws.

Based on a continuous and unimodal distribution of jaw/mouth asymmetry and the significant laboratory and field *h* ^*2*^ estimates, we suggest that mouth asymmetry of *P. microlepis* is determined by both genetic and environmental effects. The presence of juvenile individuals with already noticeable left‐ or right‐bending jaws even in early ontogenetic stages suggests that this morphological asymmetry has a genetic basis (Stewart and Albertson 2010). This asymmetry is likely to be “inborn” [not because of their feeding habit (i.e., scale‐eating behavior)] because young *P. microlepis* of less than 3 cm in standard length feed exclusively on zooplankters such as copepods (Nshombo et al. 1985). The trend that L‐L pairs tend to produce more L morphs and R‐R pairs R morphs (Table 1; Fig. 2E and F) supports the hypothesis of the genetic basis of mouth laterality. Evidence for the effects of environmental variation on the determination of mouth asymmetry derives from recent studies that found the role of “feeding environment” in facilitating morphological mouth asymmetry (Van Dooren et al. 2010; Palmer 2011; Lee et al. 2012).

Our results show that although both the field and laboratory *h* ^*2*^ estimates of mouth laterality were significant, the laboratory estimate was slightly smaller than the field estimate. *h* ^*2*^ estimates under laboratory conditions are hypothesized to be generally greater than those measured from the field, due to the assumption that the amount of environmental variance under laboratory conditions would be smaller, which decreases it relative to additive genetic variance, entailing an increase in *h* ^2^ (Mitchell‐Olds and Rutledge 1986). However, a review (Weigensberg and Roff 1996) compared the previously reported *h* ^2^ estimates (165 field and 189 laboratory estimates) and found that the field estimates are indeed higher than the laboratory estimates in general, particularly for morphological traits. Yet, in cases of *h* ^2^ estimates of the same morphological traits that have been calculated in both laboratory and field, laboratory estimates were found to be slightly higher than field estimates, but this trend was not statistically significant (Weigensberg and Roff 1996). Alternatively, the use of different mouth asymmetry estimates for wild‐caught and laboratory‐bred broods could explain the reason why the field estimate is higher than the laboratory one. The repeatability of asymmetry measurements was fairly lower for laboratory broods (*r *=* *0.8) than field broods (*r *=* *0.96), which may account for the discrepancy between laboratory and field estimates.

Here, we show that the remarkable morphological variation in populations of *P. microlepis* can be attributed to both genetic and environmental influences. These findings provide information on an issue about how morphological asymmetry evolves in general as there must be some “heritable” variation in traits for phenotypic evolution by natural (or sexual) selection to take place. Given this, a further understanding of its genetic basis would be aided by localizing/characterizing genomic locations involved in this laterality through QTL (quantitative trait loci) mapping analysis. Transcriptomic analysis on asymmetric bones for mouth laterality using a RNA‐Seq technique can be an alternative, promising approach to identify laterally differentially expressed genes and thereby advance our understanding of the genetic basis of laterality.

## Conflict of Interest

None declared.
